# A machine learning-based approach to ERα bioactivity and drug ADMET prediction

**DOI:** 10.3389/fgene.2022.1087273

**Published:** 2023-01-04

**Authors:** Tianbo An, Yueren Chen, Yefeng Chen, Leyu Ma, Jingrui Wang, Jian Zhao

**Affiliations:** ^1^ College of Network Security, Changchun University, Changchun, Jilin, China; ^2^ Institute of Education, Xiamen University, Xiamen, Fujian, China; ^3^ College of Computer Science and Technology, Changchun University, Changchun, Jilin, China

**Keywords:** machine learning, ERα bioactivity, ADMET, breast cancer, drug development

## Abstract

By predicting ERα bioactivity and mining the potential relationship between Absorption, Distribution, Metabolism, Excretion, Toxicity (ADMET) attributes in drug research and development, the development efficiency of specific drugs for breast cancer will be effectively improved and the misjudgment rate of R&D personnel will be reduced. The quantitative prediction model of ERα bioactivity and classification prediction model of Absorption, Distribution, Metabolism, Excretion, Toxicity properties were constructed. The prediction results of ERα bioactivity were compared by XGBoot, Light GBM, Random Forest and MLP neural network. Two models with high prediction accuracy were selected and fused to obtain ERα bioactivity prediction model from Mean absolute error (MAE), mean squared error (MSE) and R2. The data were further subjected to model-based feature selection and FDR/FPR-based feature selection, respectively, and the results were placed in a voting machine to obtain Absorption, Distribution, Metabolism, Excretion, Toxicity classification prediction model. In this study, 430 molecular descriptors were removed, and finally 20 molecular descriptors with the most significant effect on biological activity obtained by the dual feature screening combined optimization method were used to establish a compound molecular descriptor prediction model for ERα biological activity, and further classification and prediction of the Absorption, Distribution, Metabolism, Excretion, Toxicity properties of the drugs were made. Eighty variables were selected by the model ExtraTreesClassifier Classifie, and 40 variables were selected by the model GradientBoostingClassifier to complete the model-based feature selection. At the same time, the feature selection method based on FDR/FPR is also selected, and the three classification models obtained by the two methods are placed into the voting machine to obtain the final model. The experimental results showed that the model‘s evaluation indexes and roc diagram were excellent and could accurately predict ERα bioactivity and Absorption, Distribution, Metabolism, Excretion, Toxicity properties. The model constructed in this study has high accuracy, fast convergence and robustness, has a very high accuracy for Absorption, Distribution, Metabolism, Excretion, Toxicity and ERα classification prediction, has bright prospects in the biopharmaceutical field, and is an important method for energy conservation and yield increase in the future.

## 1 Introduction

With the development of the times, more and more people began to pay attention to their own health problems. In today’s society, breast cancer is one of the most common and lethal cancers. Estrogen receptors have a close correlation with the development of breast cancer, and according to related studies, estrogen receptor alpha (ERα) in estrogen has a strong promoting effect on the dominant characterization of breast cancer (Lempereur et al., 2016).

At present, the main treatment for breast cancer patients is to inhibit the expression of ERα gene using anti-hormone therapy, and then control the estrogen level in patients through the modulation of estrogen receptor activity, so as to achieve the inhibition of breast cancer spread and malignant trend, and gradually combine drugs and radiochemotherapy to achieve effective treatment of breast cancer (Ali and Coombes, 2002; Mohla et al., 2009; Huang et al., 2015). Drugs developed based on the corresponding compounds have been widely used in clinical treatment.

Drug R&D involves the discovery and development of new drugs, and the difference between these two stages is the determination of candidate drugs, candidate drugs represent active compounds involved in clinical research, so it is necessary to screen active compounds for drug research and development. Active compounds are compounds with certain biological or pharmacological activities obtained through various ways and methods, in the screening process, we should do research on biological activity, pharmacokinetics, toxicity analysis, etc. while synthesizing, so as to find the molecules needed for drug development. Then it is necessary to conduct tests related to biological activity and pharmacological data, that is, the quantitative structure activity relationship (QSAR) model (Bolboaca and Jäntschi, 1900; Singh et al., 2013; Ezugwu et al., 2021) of the compound. After the screening work is completed, in order to avoid the late risk of drug development (Samuel et al., 2021; Sun et al., 2022), also needs to verify whether it has ADMET (Absorption, Distribution, Metabolism, Excretion, Toxicity) properties. In the compound database, the construction vector can select compounds with excellent ADMET properties and biological activities (Deng, 2013). Mining tacit knowledge in drug data and using machine learning prediction can reduce the R&D cost of pharmaceutical processes (Guo et al., 2022), and deep learning algorithms accelerate drug target recognition efficiency (Fenglei et al., 2021). Construction of prediction models for protein hotspot residues based on machine learning algorithms can assist drug development (Hu, 2019). Further mining the activity of drugs against tumor therapeutic targets and the sensitivity of tumor cell lines can efficiently develop novel tumor drugs (Li, 2021). The ADMET classification prediction model has good performance in predicting the properties of anti-breast cancer drugs (Yaqin et al., 2022). Scholars have found that some genes as well as core TFs can evaluate the efficacy of adjuvant therapy for breast cancer, of which E2F1 can regulate MAPK signaling pathways involved in pharmaceutical processes (Ye et al., 2022). Recognizing that AI is a very effective tool in disease assessment, patient data are collected through machine learning to develop mathematical models and predict outcomes (Suh and Lee, 2017; Fu et al., 2022; Zheng et al., 2022), a large number of researchers have investigated interpretable disease diagnostic models (Casteleiro-Roca et al., 2020; Tjoa and Guan, 2021; Xu et al., 2022).

Jiang and other scholars established an easy to understand OPLS-DA model based on only two descriptors, and the accuracy of the model was as high as 93% and 79% (Matsson et al., 2007; Jiang et al., 2020). Pan and other scholars trained 79, 99 and 780 compounds respectively, and achieved high accuracy (Jiang et al., 2020). Although good results have been achieved, there is still room for optimization in terms of descriptors and the number of compounds. Based on 1974 compounds (samples) and 729 molecular descriptors (variables) provided by candidate compound data set, this paper solves the problem of low reliability caused by previous model development based on relatively small data set. In addition, the application value of the existing ADMET model in specific drug screening work is still unclear, based on this, this study, first, a dual feature screening combination method is proposed to screen data variables; then, the combination of Bagging algorithm and random forest algorithm is used to realize ERα’ Bioactivity prediction; finally, different target values are introduced to test the performance of the prediction model, so as to improve the development efficiency of breast cancer specific drugs, as well as reducing the error judgment rate of R&D personnel.

## 2 Materials and methods

### 2.1 Data acquisition

The main ways to obtain drug data include laboratories, online public publications, biochemical databases, and so on. In this paper, the optimized modeling (2021) dataset of anti-breast cancer drug candidates provided by the China Association for Science and Technology is used as the candidate compound dataset.

Taking anti-breast cancer drugs as an example to test the prediction framework for the following reasons: First, breast cancer is the largest cancer in the world, and breast cancer patients in China account for a relatively large proportion; second, researchers have accumulated a large number of R&D data of anti-breast cancer drugs over the years, laying the foundation for further machine-learning-based prediction research; third, predecessors have left sufficient literature data on breast cancer characteristic targets, which plays a guiding role in the establishment of the validation prediction framework.

### 2.2 Data processing

**FIGURE 1:**
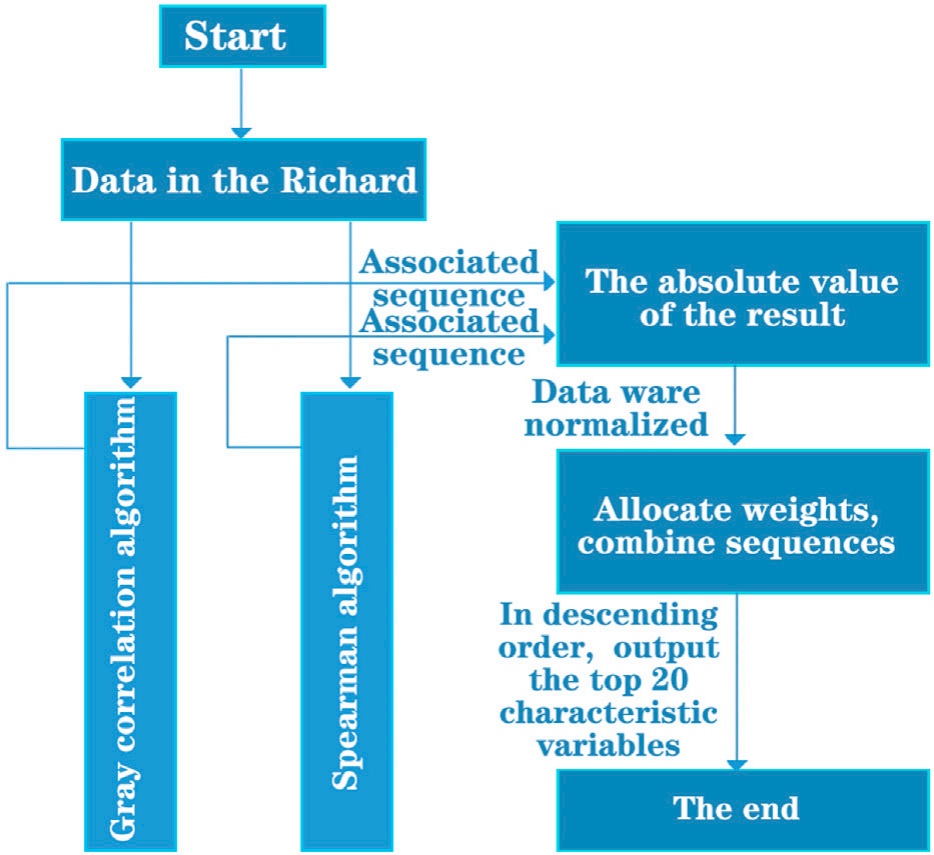
Flow chart of combined optimization method for dual feature screening.

According to the data of 1974 compounds (samples) and 729 molecular descriptors (variables) provided by the candidate compound dataset, 20 main variables were screened, which made the selected main variables have a good effect on ERα biological activity. Data preprocessing is performed first, and column variables (molecular descriptors) are cleaned. Different screening algorithms were used to analyze the distribution characteristics of the cleaned data. In order to obtain more accurate screening results, gray relational degree method and Spearman rank correlation coefficient analysis are selected to reduce the dimension of the data and data screening, and the two are further combined analysis, and a dual feature screening combined optimization method is proposed. The specific analysis is divided into k steps, and the flow chart is shown in Figure 1.

Step 1 Relevance calculation. The data were processed using Algorithm I (grey correlation analysis) and Algorithm III (Spearman rank correlation analysis) to obtain the corresponding correlation coefficient sequences $X\left(i\right)$ and $Y\left(j\right)$ .

Grey correlation analysis method, which takes the difference value between curves as the correlation degree. There are several comparison sequences (*x*_1_, *x*_2_,…, *x*_*n*_) for a reference sequence *x*_0_, correlation coefficient of each comparison sequence and reference sequence at each time (i.e., each point in the curve) *β*(*x*_*i*_) can be calculated by the following formula: *ρ* is the resolution coefficient, generally between 0 and 1, usually taken as 0.5. *Δmin* represents the second level minimum difference, *Δmax* represents the maximum difference between two levels. *Δ*_*oi*_(*k*) represents the absolute difference between each point on the comparison series *x*_*i*_ curve and each point on the reference series *x*_0_ curve. Therefore, the correlation coefficient *β*(*x*_*i*_) can also be simplified as follows:

$$\beta_{oi}=\frac{\Delta \left(\min\right)+\rho \Delta \left(\max\right)}{\Delta_{oi}\left(k\right)+\rho \Delta \left(\max\right)}$$ (1)

Because the correlation coefficient is the value of the correlation degree between the comparison sequence and the reference sequence at each time (i.e., each point in the curve), it has more than one number, and the information is too scattered to facilitate the overall comparison. Therefore, it is necessary to centralize the correlation coefficient of each time (i.e., each point in the curve) into one value, that is, to calculate its average value, as a quantitative expression of the correlation degree between the comparison sequence and the reference sequence, the formula of correlation degree *c*_*i*_ is as follows:

$$c_{i}=\frac{1}{N}\overset{N}{\underset{k=1}{\sum }}\beta_{i}\left(k\right)$$ (2)

Spearman rank correlation coefficient is generally considered as Pearson linear correlation coefficient between ranked variables, in actual calculation, there are simpler calculation methods. Assume that the original data *x*_*i*_, *y*_*i*_ has been arranged from large to small, put *x*’_*i*_, *y*’_*i*_ as the location of the original *x*_*i*_, *y*_*i*_ data after arrangement, then *d*_*i*_ = *x*’_*i*_−*y*_*’i*_ represents the difference of rank between *x*_*i*_, *y*_*i*_.

If there is no same rank, Spearman rank correlation coefficient can be expressed as:

$$spc=1-\frac{6\sum d_{i}^{2}}{n\left(n^{2}-1\right)}$$ (3)

If the same rank exists, it is necessary to calculate Pearson’s linear correlation coefficient between ranks:

$$spc=\frac{\sum_{i}\left(x_{i}-x^{\prime }\right)\left(y_{i}-y^{\prime }\right)}{\sqrt{\sum_{i}{\left(x_{i}-x^{\prime }\right)}^{2}\sum_{i}{\left(y_{i}-y^{\prime }\right)}^{2}}}$$ (4)

Step 2 Take absolute value. Because the correlation degree derived by the two algorithms may be positive or negative, but in fact the correlation degree is similar to the concept of “distance,” neither positive correlation nor negative correlation affects the judgment of the correlation size. Therefore, the correlation degree is taken as the absolute value to more clearly represent the correlation between variables. The formula is shown below.

$$\begin{array}{l} X\left(i\right)=\left|X\left(i\right)\right|\\ Y\left(j\right)=\left|Y\left(j\right)\right| \end{array}$$ (5)

Step 3 Data normalisation. As two different correlation analysis methods are used in this paper, the correlation values $X\left(i\right)$ and $Y\left(j\right)$ derived from the two algorithms need to be normalised in order to obtain the final screening results. The normalisation algorithm used in this paper is (0, 1) normalisation and the formula is shown below.

$$x_{normalization}=\frac{x-\mathrm{Min}}{\mathrm{Max}-\mathrm{Min}}$$ (6)

Step 4 Assignment of weights. By comparing the magnitude and distribution of correlation under the two analysis methods, it is approximated that the screening process of the data set by Algorithm 1 (grey correlation analysis method) can obtain a higher correlation value. Therefore, a weight of 0.6 was assigned to sequence $X\left(i\right)$ and a weight of 0.4 to sequence $Y\left(j\right)$ . By assigning weights, the final correlation degree sequence is obtained. The calculation formula is shown below.

$$Z\left(k\right)=0.6X\left(k\right)+0.4Y\left(k\right)$$ (7)

Step 5 Sorting. The normalised variable data is sorted according to the magnitude of the correlation values to produce the top 20 most significant sub-descriptors for biological activity.

### 2.3 Data modelling

**FIGURE 2:**
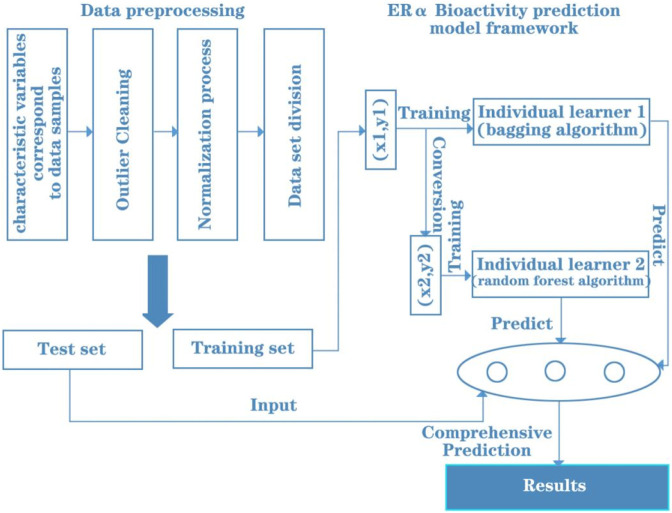
Framework of ERα bioactivity prediction model.

The 20 main variables (i.e., molecular descriptors) obtained from data processing were used to model the prediction of ERα biological activity by molecular descriptors of compounds. Data standardisation was first performed to achieve uniformity of magnitude. The dataset was then partitioned by K-fold cross-validation to achieve adequate use of the dataset to fit the prediction model. Finally, integrated learning is used to combine the Bagging and Random Forest algorithms through the Stacking method to build the entire prediction model framework. In summary, the immediate ERα bioactivity prediction model framework is shown in Figure 2.

**FIGURE 3:**
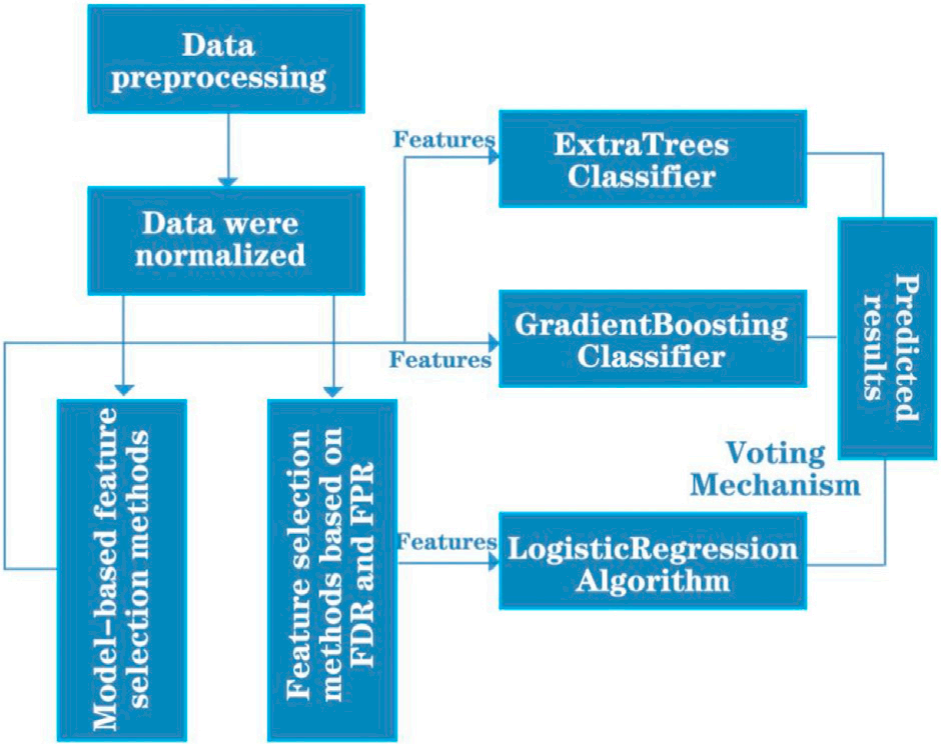
Schematic diagram of the classification prediction model framework.

In order to make further classification predictions of the ADMET properties of the drug, the original 729 molecular descriptor variables were processed for data in this paper, and a total of 300 molecular descriptor variables were retained for data analysis. After normalizing the data, the data were subjected to model-based feature selection and FDR/FPR-based feature selection, respectively. After the two model selections, the classification models obtained from the two methods were placed into a voting machine to obtain the final model, and the entire prediction model framework was constructed. In summary, the classification prediction model framework for the five specific compound classes is shown in Figure 3.

### 2.4 Evaluation of the ERα bioactivity prediction model

For the ERα bioactivity prediction model, three evaluation metrics shown in Table 1 are used in this paper.

**TABLE 1:** Evaluation metrics for regression problems.

Full name	Abbreviations	Meaning
Mean squared error	MSE	Mean square error
Mear absolute error	MAE	Mean absolute error
Coefficient of determination	$R^{2}$	Decidability factor

The mean squared error (MSE) reflects the degree of correlation between the independent and dependent variables; the MSE evaluates the degree of variation in the data.

Mean absolute error (MAE) is the average of the absolute errors and is often used to reflect the reality of the error in the predicted values.

This coefficient is often used to reflect the degree of reliability of changes in the dependent variable in a regression model. The higher the value, the better the predicted value fits the true value.

### 2.5 Evaluation of classification prediction models for ADMET properties of compounds

The experimental data was designed to reduce the rate of misclassification by drug developers, in order to ensure robustness. In this paper, five performance comparison metrics were selected: accuracy, precision, recall, F1 value, Cohen’s Kappa coefficient and ExtraTreesClassifier accuracy. The formulae for each evaluation metric are shown below.

The accuracy rate is calculated as shown in Table 2.

$$Accuracy=\frac{TP+TN}{TP+TN+FP+FN}$$ (8)

where *TP* is a positive example judged to be positive, *FP* is a negative example judged to be positive, *TN* is a negative example judged to be negative and *FN* is a positive example judged to be negative.

**TABLE 2:** The 20 most significant sub-descriptors (i.e., variables) for biological activity.

Molecular descriptors	Grey correlation	Spearman’s rank correlation coefficient	Normalised and weighted score values
MDEC-23	0.7997	0.5491	0.9999
MLogP	0.7943	0.5452	0.9872
LipoaffinityIndex	0.7954	0.5249	0.9707
CrippenLogP	0.7861	0.4738	0.9081
SwHBa	0.7895	0.4457	0.8883
nC	0.7641	0.4868	0.8821
nT6Ring	0.7809	0.4383	0.8668
n6Ring	0.7861	0.4281	0.8665
BCUTp-1h	0.7821	0.4329	0.8639
SP-5	0.7764	0.4427	0.8631
C2SP2	0.7686	0.4430	0.8499
SP-6	0.7775	0.4201	0.8444
ATSp4	0.7642	0.4444	0.8437
ATSp2	0.7601	0.4443	0.8365
ATSp5	0.7537	0.4514	0.8321
ATSp3	0.7648	0.4296	0.8312
maxsOH	0.7460	0.4619	0.8284
ATSp1	0.7550	0.4439	0.8274
nHaaCH	0.7632	0.4226	0.8221
naaCH	0.7632	0.4226	0.8221

The accuracy rate is calculated by the formula:

$$\mathrm{Pr}ecision=\frac{TP}{TP+FP}$$ (9)

The recall is calculated as:

$$Recall=\frac{TP}{TP+FN}$$ (10)

The F1 value is calculated using the formula:

$$F1=\frac{2TP}{2TP+FP+FN}$$ (11)

The Cohen’s Kappa coefficient is calculated as:

$$k=\frac{p_{0}-p_{e}}{1-p_{e}}$$ (12)

Where, *p*_0_ represents the observed compliance rate and *p*_*e*_ represents the opportunity compliance rate.

### 2.6 ROC curve

Horizontal axis FPR: 1-TNR, 1-Specificity. The larger the FPR, the more actual negative classes in the predicted positive classes. Vertical axis TPR: Sensitivity. The larger the TPR, the more actual positive classes in the predicted positive classes. The closer the ROC curve is to the (0, 1) point, the more it deviates from the 45° diagonal, the better.

## 3 Results

### 3.1 Dataset description

The optimized modelling of anti-breast cancer drug candidates (2021) dataset provided by CCSA was used. The 729 molecular descriptors of 1974 compounds in it were used as dependent variables to find the molecular descriptors among them that could significantly affect ERα activity as feature variables for subsequent questions. Inevitably, there are some anomalies in the data used as dependent variables, which will interfere with the selection of the characteristic variables and have a collateral negative impact on the solution of the subsequent problem. For example, a large proportion of the numerical columns of the molecular descriptors have null (0) or almost null (0) values.

**FIGURE 4:**
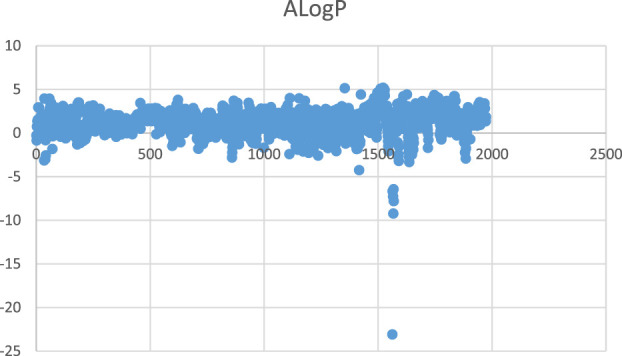
Scatter plot of ALogP.

If there are too many null values, the data reliability of the variable will be low, and it is considered that the molecular descriptors corresponding to these data are unlikely to become feature variables and will waste arithmetic power in the subsequent screening of the feature vectors. Therefore, in this step, the numerator descriptors with 95% of the data items being null are eliminated. The data visualisation also revealed some outliers in the data, and a scatter plot of one of the columns is shown in Figure 4.

The raw data was normalised and after noise and dimensionality reduction to form the experimental dataset. Using the KS test, the study found that the data samples did not satisfy a normal distribution, which in turn led to the use of an outlier treatment based on box plot analysis.

**FIGURE 5:**
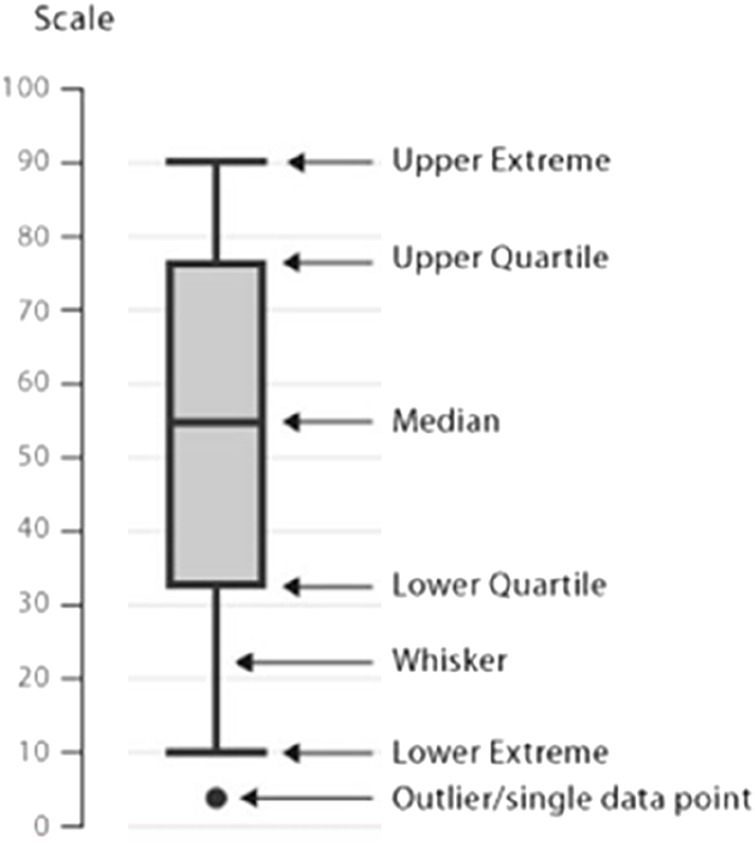
Schematic diagram of the box line diagram.

After sorting each column of data from smallest to largest, the interval between the values of the first and third quartiles is taken as the acceptable range, where the upper bound is the third quartile—IQR (IQR = third quartile–first quartile) and the lower bound is the first quartile—IQR, and numbers outside this value area are considered outliers. These outliers are taken to correspond to the element values, with outliers over the upper bound being taken to the upper bound and those over the lower bound being taken to the lower bound, otherwise they remain as they are. Then go back to Step 1 and clear the column with the higher number of null values. As shown in Figure 5.

Through the above data processing, a total of 430 molecular descriptors were removed. The final 20 molecular descriptors (i.e., variables) with the most significant impact on bioactivity as a result of the dual feature screening combined optimisation method are shown in Table 3.

**TABLE 3:** Descriptions of null-valued columns (e.g., nB).

Count	1974
Mean	0
Std	0
Min	0
25%	0
50%	0
75%	0
Max	0

**FIGURE 6:**
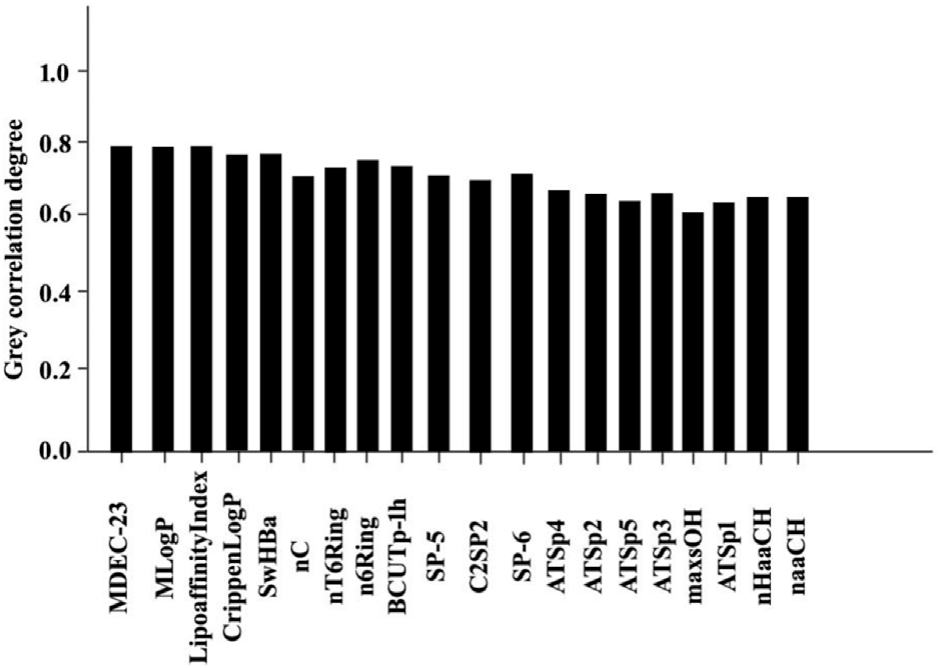
Statistical results of 20 bioactive molecules based on Grey correlation analysis.

**FIGURE 7:**
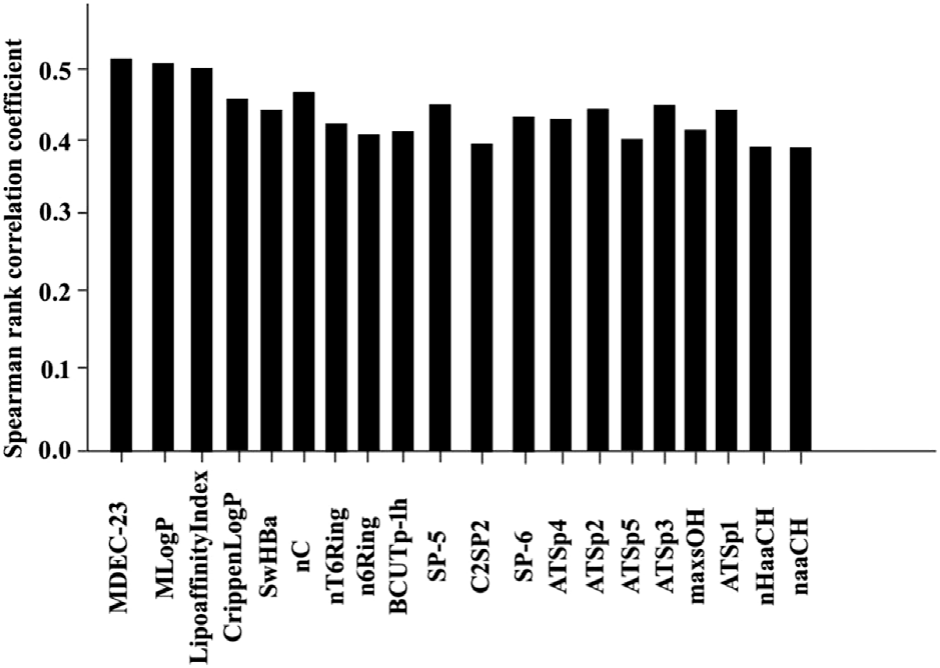
Statistical results of 20 bioactive molecules based on Spearman rank correlation coefficient analysis.

The results of the Spearman rank correlation coefficient processing for this study are shown in Figures 6, 7

### 3.2 Data set variable screening combination optimisation

The 20 features derived from the dual feature screening combination optimisation method, Spearman’s algorithm and the grey correlation algorithm were substituted into the correlation regression model and the MAE, MSE and R^2^ were used to evaluate the advantages and disadvantages of the three methods. The table below shows the results of the above experiments. From the data in the table, it is analysed that the dual feature screening combination optimisation method used in this paper has better results. As shown in Table 4.

**TABLE 4:** Evaluation data of the dual feature screening combination optimisation method.

Weighted score	MAE	MSE	R^2^
MLPRegressor	1.3723	3.2465	−0.6000
GradientBoostingRegressor	0.6828	0.8280	0.5900
RandomForestRegressor	0.6104	0.6873	0.6607
AdaBoostRegressor	0.8500	1.1100	0.4500

### 3.3 Model performance

#### 3.3.1 ERα bioactivity prediction model evaluation

**FIGURE 8:**
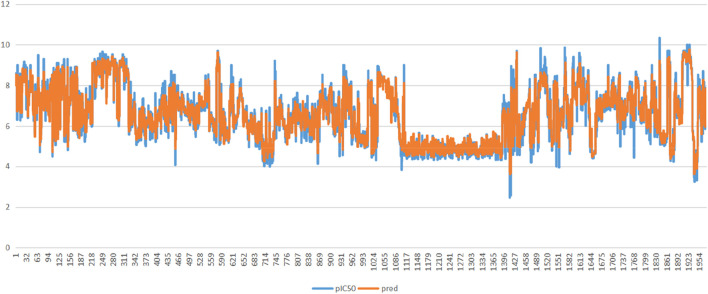
Feature variables and pIC50 fit results.

By analysing the various algorithm evaluation metrics, we found that the MLP performed poorly regardless of the metrics, and the r^2^ value of AdaBoostRegressor was consistently below 0.5, so it was also out of our selection range. The final model is the result of fusing the random forest and GradientBoostingRegresso**r** after tuning the parameters through a grid search. As shown in Figure 8.

The results in Table 5 show that the model after stacking performs better than the original two models in all three scoring metrics, and also that this model is realistic in the field of machine learning when the R^2^ value is greater than 0.7 and the fitted function is more realistic.

**TABLE 5:** Evaluation metric values for each algorithm.

	MAE	MSE	R^2^
GradientBoostingRegressor	0.6732	0.7927	0.6087
RandomForestRegressor	0.6180	0.6752	0.6600
Stacking	0.5940	0.6436	0.7002

#### 3.3.2 Evaluation of classification prediction models for the ADMET properties of compounds

For the classification prediction models of the ADMET properties of compounds, the results of the evaluation of the five target value related classification models are given below. It can be seen that in most cases, the model set up in this paper prevails. Of course, the effect is not significant due to the inherently low scores of the initial models. As shown in Table 6.

**TABLE 6:** Evaluation of classification models for each algorithm with CYP3A4 as the target value.

Algorithms	Accuracy	Accuracy	Recall rate	F1 value	Cohen’s Kappa coefficient
LogisticRegression	0.9367	0.9626	0.9529	0.9577	0.8321
ExtraTreesClassifier	0.9367	0.9564	0.9596	0.9580	0.8298
RandomForestClassifier	0.9468	0.9600	0.9697	0.9648	0.8560
Integrated learning models based on Stacking methods	0.9538	0.9668	0.9581	0.9634	0.8816

**FIGURE 9:**
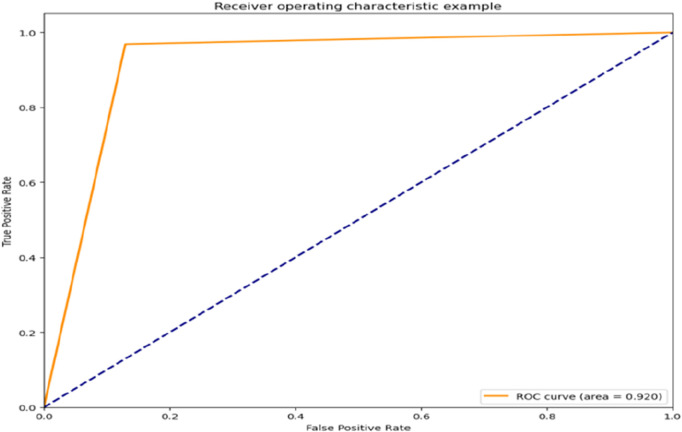
Classification model roc curve with hERG as the target value.

**FIGURE 10:**
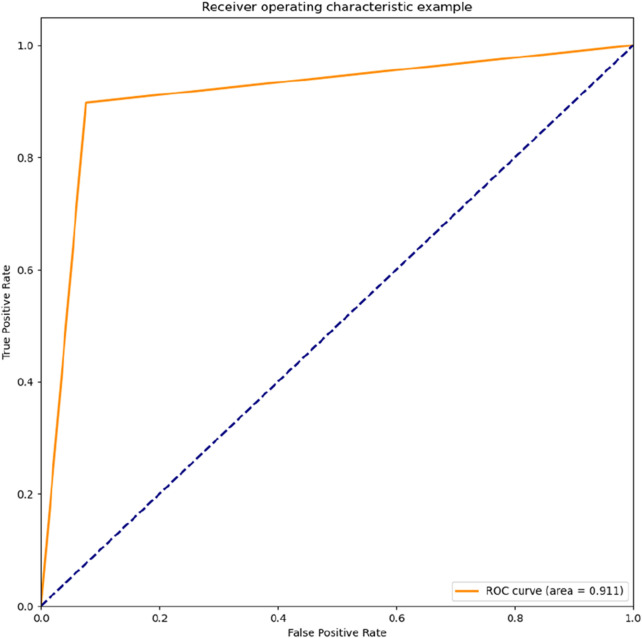
Classification model roc curve with MN as the target value.

In the following results, the roc diagram about the two toxicity indicators is given. Figures 9, 10, it can be seen that the classification effect for these two indicators is good.

The resulting three classification models were placed into the voting machine, which is the final model. Five final models were obtained on five validation sets with accuracy: 0.92,0.95,0.91,0.76,0.96; F1 values: 0.91,0.96,0.92,0.80,0.97 respectively.

## 4 Discussion

In today‘s society, breast cancer is the most common and lethal cancer and is the leading cause of cancer death in women (Desantis et al., 2015), and related studies have confirmed that breast cancer is genetically risky (Lilyquist et al., 2018). The expression of estrogen receptor ERα is closely related to the development of breast cancer, and this gene is involved in the proliferation and differentiation of breast cancer cells (Geng, 2016). Currently ERα is considered an important target for the treatment of breast cancer (Xu, 2018). Researchers have found that the ADMET module ensures that the corresponding compound becomes a candidate with good pharmacokinetic properties and safety (Dejun et al., 2018). Thus, machine learning can effectively reduce the cost of drug research and development and improve the stability and accuracy of prediction models (Wang et al., 2006). Scholars have determined that the optimal cut-off values for E2 and FSH in serum can assess CIA in breast cancer populations in southern China (Yang et al., 2022). Estrogen receptors alpha (ERα) in estrogen has been found to be an important target for the treatment of breast cancer (Chang et al., 2013).

We collected data on anti-breast cancer drug candidates provided by the China Association for Science and Technology, which provided 974 compounds (samples) and 729 molecular descriptors (variables) information. The dataset is therefore highly representative and generalizable. In this study, random forest algorithm and Bagging regression algorithm were used to construct a quantitative prediction model of ERα bioactivity by compounds, and two model selection features, ExtraTreesClassifier and GradientBoostingClassifier, were selected. According to the actual situation, taking into account the properties of target value, fdr-based method is adopted to improve F1 value in feature selection. At the same time, the feature selection method based on model also has high accuracy. Both vote to ensure the accuracy of the final model.

It is shown that the weighted scores obtained by grey correlation and Spearman coefficient are more suitable for the data distribution characteristics of anti-breast cancer candidates. In this study, 20 molecular descriptors with strong antagonistic effects were selected from 729 molecular descriptors. These are MDEC-23, MLogP, LipoaffinityIndex, etc. Random forest algorithm and Bagging regression algorithm were selected to construct the model, and then Stacking model fusion method was used to establish the prediction model framework. The model showed good performance in MAE, MSE and three scoring indexes after Stacking, and the predicted result value of the model on the validation set was 0.7, which proved that the model could accurately predict. According to the classification prediction model of ADMET properties of compounds, two feature selection methods were used to screen the features, and three classification models obtained by the two methods were placed into the voting machine to obtain high accuracy and F1 values. Therefore, the model established in this study is of great significance in assisting prediction of ERα biological activity and improving the development efficiency of specific drugs for breast cancer.

Despite the good results, there are limitations in this study. In future anti-breast cancer drug screening efforts, this study should attempt to collect more datasets for machine learning training, draw on expert opinion, and continuously optimise the feature generation tools to improve the accuracy and stability of the model. Through the interdisciplinary collaboration between machine learning and biopharmaceuticals, we can reduce the cost of pharmaceuticals, reduce the error rate of drug developers, and provide a reference for similar drug development work.

## 5 Conclusion

A quantitative prediction model for ERα bioactivity and a classification prediction model for ADMET properties of compounds were developed, which can assist in the development of specific drugs for breast cancer. With the high accuracy of the models, the cost of drug development and the rate of misclassification by developers can be effectively reduced.

## Data Availability

The original contributions presented in the study are included in the article/Supplementary Material, further inquiries can be directed to the corresponding author.
