# Supplementary material for: A machine learning-based approach to ERα bioactivity and drug ADMET prediction
Source: Front Genet. 2023 Jan 4;13:1087273. doi: 10.3389/fgene.2022.1087273 (PMC9845410; doi:10.3389/fgene.2022.1087273)
Supplement: Supplementary file 1 [file Table1.docx]

Supplementary Table 1: Evaluation data for Spearman's algorithm

|  | MAE | MSE | R2 |
| --- | --- | --- | --- |
| MLPRegressor | 0.7686 | 1.0000 | 0.5000 |
| GradientBoostingRegressor | 0.7120 | 0.8600 | 0.5700 |
| RandomForestRegressor | 0.6370 | 0.7473 | 0.6300 |
| AdaBoostRegressor | 0.8800 | 1.1400 | 0.4300 |
