# Supplementary material for: A machine learning-based approach to ERα bioactivity and drug ADMET prediction
Source: Front Genet. 2023 Jan 4;13:1087273. doi: 10.3389/fgene.2022.1087273 (PMC9845410; doi:10.3389/fgene.2022.1087273)
Supplement: Supplementary file 2 [file DataSheet1.ZIP › ┐╣╥╚╧┘░⌐║≥╤í╥⌐╬∩╡─╙┼╗»╜¿─ú.docx]

2021年中国研究生数学建模竞赛D题

**抗乳腺癌候选药物的优化建模**

**一、背景介绍**

乳腺癌是目前世界上最常见，致死率较高的癌症之一。乳腺癌的发展与雌激素受体密切相关，有研究发现，雌激素受体α亚型（Estrogen receptors alpha, ERα）在不超过10%的正常乳腺上皮细胞中表达，但大约在50%-80%的乳腺肿瘤细胞中表达；而对ERα基因缺失小鼠的实验结果表明，ERα确实在乳腺发育过程中扮演了十分重要的角色。目前，抗激素治疗常用于ERα表达的乳腺癌患者，其通过调节雌激素受体活性来控制体内雌激素水平。因此，ERα被认为是治疗乳腺癌的重要靶标，能够拮抗ERα活性的化合物可能是治疗乳腺癌的候选药物。比如，临床治疗乳腺癌的经典药物他莫昔芬和雷诺昔芬就是ERα拮抗剂。

目前，在药物研发中，为了节约时间和成本，通常采用建立化合物活性预测模型的方法来筛选潜在活性化合物。具体做法是：针对与疾病相关的某个靶标（此处为ERα），收集一系列作用于该靶标的化合物及其生物活性数据，然后以一系列分子结构描述符作为自变量，化合物的生物活性值作为因变量，构建化合物的定量结构-活性关系（Quantitative Structure-Activity Relationship, QSAR）模型，然后使用该模型预测具有更好生物活性的新化合物分子，或者指导已有活性化合物的结构优化。

一个化合物想要成为候选药物，除了需要具备良好的生物活性（此处指抗乳腺癌活性）外，还需要在人体内具备良好的药代动力学性质和安全性，合称为ADMET（Absorption吸收、Distribution分布、Metabolism代谢、Excretion排泄、Toxicity毒性）性质。其中，ADME主要指化合物的药代动力学性质，描述了化合物在生物体内的浓度随时间变化的规律，T主要指化合物可能在人体内产生的毒副作用。一个化合物的活性再好，如果其ADMET性质不佳，比如很难被人体吸收，或者体内代谢速度太快，或者具有某种毒性，那么其仍然难以成为药物，因而还需要进行ADMET性质优化。为了方便建模，本试题仅考虑化合物的5种ADMET性质，分别是：1）小肠上皮细胞渗透性（Caco-2），可度量化合物被人体吸收的能力；2）细胞色素P450酶（Cytochrome P450, CYP）3A4亚型（CYP3A4），这是人体内的主要代谢酶，可度量化合物的代谢稳定性；3）化合物心脏安全性评价（human Ether-a-go-go Related Gene, hERG），可度量化合物的心脏毒性；4）人体口服生物利用度（Human Oral Bioavailability, HOB），可度量药物进入人体后被吸收进入人体血液循环的药量比例；5）微核试验（Micronucleus，MN），是检测化合物是否具有遗传毒性的一种方法。

**二、数据集介绍及建模目标**

本试题针对乳腺癌治疗靶标ERα，首先提供了1974个化合物对ERα的生物活性数据。这些数据包含在文件“ERα_activity.xlsx”的training表（训练集）中。training表包含3列，第一列提供了1974个化合物的结构式，用一维线性表达式SMILES（**S**implified **M**olecular **I**nput **L**ine **E**ntry **S**ystem）表示；第二列是化合物对ERα的生物活性值（用IC_50_表示，为实验测定值，单位是nM，值越小代表生物活性越大，对抑制ERα活性越有效）；第三列是将第二列IC_50_值转化而得的pIC_50_（即IC_50_值的负对数，该值通常与生物活性具有正相关性，即pIC_50_值越大表明生物活性越高；实际QSAR建模中，一般采用pIC_50_来表示生物活性值）。该文件另有一个test表（测试集），里面提供有50个化合物的SMILES式。

其次，在文件“Molecular_Descriptor.xlsx”的training表（训练集）中，给出了上述1974个化合物的729个分子描述符信息（即自变量）。其中第一列也是化合物的SMILES式（编号顺序与上表一样），其后共有729列，每列代表化合物的一个分子描述符（即一个自变量）。化合物的分子描述符是一系列用于描述化合物的结构和性质特征的参数，包括物理化学性质（如分子量，LogP等），拓扑结构特征（如氢键供体数量，氢键受体数量等），等等。关于每个分子描述符的具体含义，请参见文件“分子描述符含义解释.xlsx”。同样地，该文件也有一个test表，里面给出了上述50个测试集化合物的729个分子描述符。

最后，在关注化合物生物活性的同时，还需要考虑其ADMET性质。因此，在文件“ADMET.xlsx”的training表（训练集）中，提供了上述1974个化合物的5种ADMET性质的数据。其中第一列也是表示化合物结构的SMILES式（编号顺序与前面一样），其后5列分别对应每个化合物的ADMET性质，采用二分类法提供相应的取值。Caco-2：‘1’代表该化合物的小肠上皮细胞渗透性较好，‘0’代表该化合物的小肠上皮细胞渗透性较差；CYP3A4：‘1’代表该化合物能够被CYP3A4代谢，‘0’代表该化合物不能被CYP3A4代谢；hERG：‘1’代表该化合物具有心脏毒性，‘0’代表该化合物不具有心脏毒性；HOB：‘1’代表该化合物的口服生物利用度较好，‘0’代表该化合物的口服生物利用度较差；MN：‘1’代表该化合物具有遗传毒性，‘0’代表该化合物不具有遗传毒性。同样地，该文件也有一个test表，里面提供有上述50个化合物的SMILES式（编号顺序同上）。

**建模目标：**根据提供的ERα拮抗剂信息（1974个化合物样本，每个样本都有729个分子描述符变量，1个生物活性数据，5个ADMET性质数据），构建化合物生物活性的定量预测模型和ADMET性质的分类预测模型，从而为同时优化ERα拮抗剂的生物活性和ADMET性质提供预测服务。

**三、需解决问题**

**问题1.** 根据文件“Molecular_Descriptor.xlsx”和“ERα_activity.xlsx”提供的数据，针对1974个化合物的729个分子描述符进行变量选择，根据变量对生物活性影响的重要性进行排序，并给出前20个对生物活性最具有显著影响的分子描述符（即变量），并请详细说明分子描述符筛选过程及其合理性。

**问题2.** 请结合问题1，选择不超过20个分子描述符变量，构建化合物对ERα生物活性的定量预测模型，请叙述建模过程。然后使用构建的预测模型，对文件“ERα_activity.xlsx”的test表中的50个化合物进行IC_50_值和对应的pIC_50_值预测，并将结果分别填入“ERα_activity.xlsx”的test表中的IC50_nM列及对应的pIC50列。

**问题3.** 请利用文件“Molecular_Descriptor.xlsx”提供的729个分子描述符，针对文件“ADMET.xlsx”中提供的1974个化合物的ADMET数据，分别构建化合物的Caco-2、CYP3A4、hERG、HOB、MN的分类预测模型，并简要叙述建模过程。然后使用所构建的5个分类预测模型，对文件“ADMET.xlsx”的test表中的50个化合物进行相应的预测，并将结果填入“ADMET.xlsx”的test表中对应的Caco-2、CYP3A4、hERG、HOB、MN列。

**问题4.** 寻找并阐述化合物的哪些分子描述符，以及这些分子描述符在什么取值或者处于什么取值范围时，能够使化合物对抑制ERα具有更好的生物活性，同时具有更好的ADMET性质（给定的五个ADMET性质中，至少三个性质较好）。

**附件：**

附件一：ERα_activity.xlsx

附件二：Molecular_Descriptor.xlsx

附件三：分子描述符含义解释.xlsx

附件四：ADMET.xlsx
