# Supplementary material for: A machine learning-based approach to ERα bioactivity and drug ADMET prediction
Source: Front Genet. 2023 Jan 4;13:1087273. doi: 10.3389/fgene.2022.1087273 (PMC9845410; doi:10.3389/fgene.2022.1087273)
Supplement: Supplementary file 3 [file Table2.docx]

Supplementary Table 2: Evaluation data for the grey correlation algorithm

|  | MAE | MSE | R2 |
| --- | --- | --- | --- |
| MLPRegressor | 3.3820 | 17.7500 | -7.7000 |
| GradientBoostingRegressor | 0.6737 | 0.7940 | 0.6080 |
| RandomForestRegressor | 0.6150 | 0.6739 | 0.6673 |
| AdaBoostRegressor | 0.8370 | 1.0650 | 0.4740 |
