# Supplementary material for: A machine learning-based approach to ERα bioactivity and drug ADMET prediction
Source: Front Genet. 2023 Jan 4;13:1087273. doi: 10.3389/fgene.2022.1087273 (PMC9845410; doi:10.3389/fgene.2022.1087273)
Supplement: Supplementary file 4 [file Table5.docx]

**Supplementary Table 5: Evaluation of classification models for each algorithm with HOB as the target value**

| Algorithms | Accuracy | Accuracy | Recall rate | F1 value | Cohen’s Kappa Coefficient |
| --- | --- | --- | --- | --- | --- |
| LogisticRegression | 0.8532 | 0.6979 | 0.6979 | 0.6979 | 0.6009 |
| ExtraTreesClassifier | 0.8684 | 0.9500 | 0.7292 | 0.7292 | 0.6422 |
| RandomForestClassifier | 0.8633 | 0.7100 | 0.7396 | 0.7245 | 0.6336 |
| Integrated learning models based on Stacking methods | 0.8938 | 0.7468 | 0.7581 | 0.7534 | 0.6616 |
