# Supplementary material for: A machine learning-based approach to ERα bioactivity and drug ADMET prediction
Source: Front Genet. 2023 Jan 4;13:1087273. doi: 10.3389/fgene.2022.1087273 (PMC9845410; doi:10.3389/fgene.2022.1087273)
Supplement: Supplementary file 5 [file Table3.docx]

**Supplementary Table 3: Evaluation of classification models for each algorithm with Caco-2 as the target value**

| Algorithms | Accuracy | Accuracy | Recall rate | F1 value | Cohen’s Kappa Coefficient |
| --- | --- | --- | --- | --- | --- |
| LogisticRegression | 0.9139 | 0.8868 | 0.8981 | 0.8924 | 0.8207 |
| ExtraTreesClassifier | 0.8886 | 0.8742 | 0.8408 | 0.8571 | 0.7659 |
| RandomForestClassifier | 0.8937 | 0.8758 | 0.8535 | 0.8645 | 0.7770 |
| Integrated learning models based on Stacking methods | 0.9238 | 0.9168 | 0.9081 | 0.9134 | 0.8516 |
