# Supplementary material for: A machine learning-based approach to ERα bioactivity and drug ADMET prediction
Source: Front Genet. 2023 Jan 4;13:1087273. doi: 10.3389/fgene.2022.1087273 (PMC9845410; doi:10.3389/fgene.2022.1087273)
Supplement: Supplementary file 6 [file Table4.docx]

**Supplementary Table 4: Evaluation of classification models for each algorithm with hERG as the target value**

| Algorithms | Accuracy | Accuracy | Recall rate | F1 value | Cohen’s Kappa Coefficient |
| --- | --- | --- | --- | --- | --- |
| LogisticRegression | 0.8759 | 0.8800 | 0.9000 | 0.8899 | 0.7479 |
| ExtraTreesClassifier | 0.9114 | 0.8970 | 0.9500 | 0.9227 | 0.8191 |
| RandomForestClassifier | 0.9063 | 0.8861 | 0.9545 | 0.9190 | 0.8083 |
| Integrated learning models based on Stacking methods | 0.9138 | 0.9068 | 0.9681 | 0.9234 | 0.8316 |
