# Supplementary material for: A machine learning-based approach to ERα bioactivity and drug ADMET prediction
Source: Front Genet. 2023 Jan 4;13:1087273. doi: 10.3389/fgene.2022.1087273 (PMC9845410; doi:10.3389/fgene.2022.1087273)
Supplement: Supplementary file 7 [file Table6.docx]

**Supplementary Table 6: Evaluation of classification models for each algorithm with MN as the target value**

| Algorithms | Accuracy | Accuracy | Recall rate | F1 value | Cohen’s Kappa Coefficient |
| --- | --- | --- | --- | --- | --- |
| LogisticRegression | 0.9418 | 0.9521 | 0.9686 | 0.9603 | 0.8513 |
| ExtraTreesClassifier | 0.9570 | 0.9500 | 0.9930 | 0.9710 | 0.8875 |
| RandomForestClassifier | 0.9519 | 0.9467 | 0.9895 | 0.9676 | 0.8742 |
| Integrated learning models based on Stacking methods | 0.9638 | 0.9568 | 0.9932 | 0.9734 | 0.8916 |
